# Editorial: Impact of blood flow restriction device features and methodological considerations on acute- and longitudinal responses to blood flow restricted exercise, volume II

**DOI:** 10.3389/fspor.2026.1981753

**Published:** 2026-10-06

**Authors:** Nicholas Rolnick, Tim Werner, Matthew Clarkson, Luke Hughes

**Affiliations:** 1 The BFR PROS, New York City, NY, United States; 2 Department of Exercise Science, Salisbury University, Salisbury, MD, United States; 3 Institute for Health and Sport, Victoria University, Melbourne, VIC, Australia; 4 Department of Sport, Exercise and Rehabilitation, Northumbria University, Newcastle upon Tyne, United Kingdom

**Keywords:** arterial occlusion pressure, BFR training, blood flow restriction, exercise science, rehabilitation

Volume I of this Research Topic established a foundation for understanding how device features and methodological variables associated with blood flow restriction (BFR) exercise can impact physiological and perceptual responses. Volume II continues this work across three domains: the stability and determination of limb occlusion pressure (LOP, also referred to as arterial occlusion pressure), the acute and chronic impact of autoregulation as a BFR cuff feature, and the efficacy of BFR across diverse populations, prescriptions, and exercise contexts. The 12 articles within this Research Topic highlight the continued maturation of the field, focusing on the short- and long-term impact of the methods of application and prescription that may meaningfully alter BFR's effects.

Bielitzki et al., Rolnick et al., and Alves et al. explored how LOP is measured in practice, focusing on different aspects of the pressure determination process. Bielitzki et al. investigated whether LOP could be impacted by simultaneous bilateral measurement in multiple body positions compared with sequential unilateral assessments. Their findings indicate that unilateral LOP determination could be sufficient as a substitute for bilateral pressure application in healthy participants, offering a potentially time-saving approach to calibrating the training pressures for BFR exercise without compromising accuracy. Rolnick et al. sought to determine the reliability of LOP assessment using the Delfi Personalized Tourniquet System (PTS) in sitting and standing in two independent cohorts. The findings showed significant difficulties in LOP assessment in weightbearing positions, challenging the feasibility of using this system outside of supine as well as practicalities that may prohibit users of this system from assessing LOP in the position of exercise. Alves et al. investigated how limb composition related to LOP assessment and whether limb composition is a meaningful variable to consider in algorithms adopted for non-doppler based limb circumference LOP formulas. Their findings suggest that limb composition meaningfully impacts LOP and the results challenge commonly used practices of estimating BFR training pressures based upon limb circumference alone.

The second theme within this special topic described the acute and chronic impact of autoregulation as a BFR cuff feature. Moghaddam et al. compared autoregulated and non-autoregulated BFR pressures during a single-leg wall squat exercise using the Delfi PTS in healthy participants and investigated acute physiological/neuromuscular responses in the vastus medialis obliquus muscle. They found broadly comparable responses between the two pressure regulation settings, indicating that regulation of applied pressure may not be necessary to maximize physiological and neuromuscular responses during non-failure BFR resistance exercise. Akçay et al. performed the first longitudinal resistance-training trial that sought to determine whether there were any muscle hypertrophy and strength differences between regulated and unregulated pressures over 8 weeks in a within-subject design using the Fitcuffs 4.0 BFR cuffs. Their findings extend on the acute findings of Moghaddam et al. to a chronic training context indicating that the autoregulatory feature of a BFR cuff may not provide additional benefit on muscle-related outcomes that justify its higher price point. These two studies provide practical takeaways for the BFR practitioner, offering initial insights into features that may not necessarily be required for optimizing acute or longitudinal responses to BFR exercise.

The third theme within this special topic is showcasing BFR's broad efficacy across diverse populations, prescriptions, and exercise contexts. These papers highlight the real benefits of BFR against low- and high-load comparators but also illuminate that some of the uneven benefits observed may be due to methodological aspects of the BFR application and implementation parameters across different studies. For example, He et al.'s review on BFR resistance training modalities in athletes outlines the mechanisms of BFR-induced adaptations as well as the common application schemes employed in the literature, providing a conceptual framework for classifying BFR training approaches in athletic contexts. Brown et al. aimed to determine differences between continuous and intermittent BFR pressure applications in low-intensity aerobic exercise. Their findings show different acute cardiovascular, perceptual, and muscle oxygenation responses between pressure approaches, offering practical guidance on programming with BFR in populations with lower tolerances to occlusive-based exercise. Cerqueira et al. performed a pilot trial in knee osteoarthritis patients that compared occlusion pressure and blood pressure responses over 9 weeks between low-intensity (10% 1RM) and moderate-intensity (60% 1RM) exercise. They found no changes in blood pressure or occlusion pressure over the trial period, indicating the stability of both and supporting less frequent LOP assessments within this population, potentially reducing the burden of BFR's implementation over a training period by reducing the number of assessments performed. Xia et al. performed a pilot study looking at whether a 4-week exercise program performed with BFR could positively affect task-related surface electromyography activation and functional performance transfer vs. a control exercise. Their findings indicate a short-term BFR program may enhance neuromuscular efficiency before functional gains emerge, highlighting that the duration and outcomes selected for BFR exercise trials may significantly influence whether meaningful changes are observed or not. Wang et al. compared 6 weeks of unilateral low-load BFR training against high-load non-BFR training using Bulgarian split-squats in resistance-trained men, looking to determine strength, hypertrophy, and performance-related outcomes in addition to cross-education. Their findings agree with the literature at large, further supporting low-load BFR as an alternative to high-load exercise, as no differences were observed in any outcome measures. Ahmad et al. performed a single-blind, two-arm randomized 6-week training trial in inactive university-aged adults. Each group performed an identical low-load multi-component exercise program including sprints, jumps, squats, limb flexion and extension, pull-ups, curls, and grip work, increasing from 30% 1RM to 40% 1RM using 50% LOP. They found BFR produced significantly greater gains in most outcome measures (10 of 18), indicating the effectiveness of integrating BFR into low-load exercise paradigms. Qu et al. looked at the impact of a low-load BFR resistance training program on lower-limb morphology and sport-relevant functional performance in male collegiate table tennis athletes. Their findings extend the evidence on BFR training to a trained, understudied athletic population, supporting its use during in-season training or supplemental conditioning where reduced joint loading or systemic stress is desired.

The papers in this special topic continue to highlight the growth of BFR in exercise science, rehabilitation, and sports performance, and further examine how the method of application, assessment, and the technologies embedded within the cuff itself affect acute and long-term responses. As BFR research continues to grow, we call on researchers to pay close attention to how these methodological features may alter the BFR stimulus, and to adopt sound reporting practices for all trials (1).
